# Novel treatment setup for urethral carcinoma radiotherapy: A complete response case report

**DOI:** 10.1002/ccr3.3329

**Published:** 2020-09-07

**Authors:** Seyed Hadi Molana, Aram Rostami, Benyamin Khajetash, Farshid Arbabi Kalati, Asieh Tavakol, Hasan Zandi, Anita Ghaderzadeh, Mahmoudreza Akbari

**Affiliations:** ^1^ Department of Radiation Oncology Roshana Cancer Institute Tehran Iran; ^2^ Department of Medical Physics Roshana Cancer Institute Tehran Iran; ^3^ Department of Medical Physics School of Medicine Iran University of Medical Sciences Tehran Iran; ^4^ Therapy Level Laboratory Secondary Standard Dosimetry Laboratory (SSDL) Karaj Iran

**Keywords:** bolus, penis holder, silicon pellets, testicular shield, urethral cancer

## Abstract

A homemade personalized penis holder can provide the reproducibility of the penis during urethra carcinoma (UC) radiotherapy.

## Graphical abstract

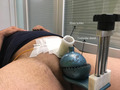

## 1 INTRODUCTION

This study offers a new treatment setup for external beam radiation therapy (EBRT) of the urethra carcinoma (UC) with reproducible supine technique obtained by homemade and personalized immobilization penis holder that at the same time, radiation dose to testicle can be reduced using testicular shield.

Urethra carcinoma (UC) is a rare form of urological cancers with a poor prognosis.^1^ In overall, UC makes up <1% of the total incidence of all malignant tumors.^2^ Although this malignancy occurs in both men and women, the incidence of UC is approximately three times more in men than in women. As reported, the age‐standardized rate (ASR) for UC is 1.6/million in men and 0.6/million in women.^3^ Up to this date, owing to the rarity of UC, no prospective studies have been performed to determine the best treatment outcomes. Therefore, information about the treatment of UC and treatment outcomes is derived from retrospective studies or case series. As a consequence, an individualized treatment approach is adopted for this malignancy. The combination of radiation therapy and chemotherapy using 5‐FU and Mitomycin‐C has been reported for locally advanced UC.^4^, ^5^, ^6^

In overall, the reproducibility of patient and target position between computed tomography (CT) planning and each treatment fraction is very important.^7^, ^8^, ^9^ In radiotherapy for the male UC, treatment setup is considered as a main challenging issue owing to the variability in the penile position. Any change in the anatomical position of the penis can reduce therapeutic ratio. The concept of radiotherapy for UC is similar to penile cancer radiotherapy. Therefore, external beam radiation therapy (EBRT) for UC is performed in the supine position. In addition, a wax block is used to support penis and spare surrounding normal tissues. There is a central cavity into wax block that encased the penis vertically.^6^, ^10^ It should be noted, however, that there is some limitation in the aforementioned technique such as lack of availability of wax block in some centers and high median dose received by testis. To this end, we offer a new treatment setup for the UC EBRT with reproducible supine technique obtained by homemade and personalized immobilization penis holder, and at the same time, it can reduce radiation dose to testicle using testicular shield.

## 2 CASE PRESENTATION

Our patient is a 30‐year‐old Asian male who had urethral obstruction, resulted in the inability to urinate. Hence, a suprapubic catheter was placed to drain urine. The patient underwent urethra biopsy by an expert urologist. A poorly differentiated urethral adenocarcinoma with invasion to underlying smooth muscle and cavernous body (stage III, pT3cN0) has been indicated by tissue biopsy. In addition, the patient underwent chest‐abdomen‐pelvic CT with no evidence of metastatic disease. The patient underwent radiotherapy in two phases. In the first phase, the patient received a dose of 50 Gy (25 × 2 Gy) to prior prostatic urethra, whole penile shaft, bladder, prostatic urethral, and pelvic lymph nodes. In the second phase, a boost dose of 10 Gy (5 × 2 Gy) was delivered to the penile shaft and prostatic urethral. The patient received five cycles of concurrent chemotherapy consisting of paclitaxel and cisplatin.

The median follow‐up time was 25 months. Complete response and local control were evaluated. Following radiotherapy, the patient reported no urinary problems and he returned he came back to normal; however, he had penile numbness and penile skin erythematic.

### 2.1 Simulation and immobilization

**FIGURE 1:**
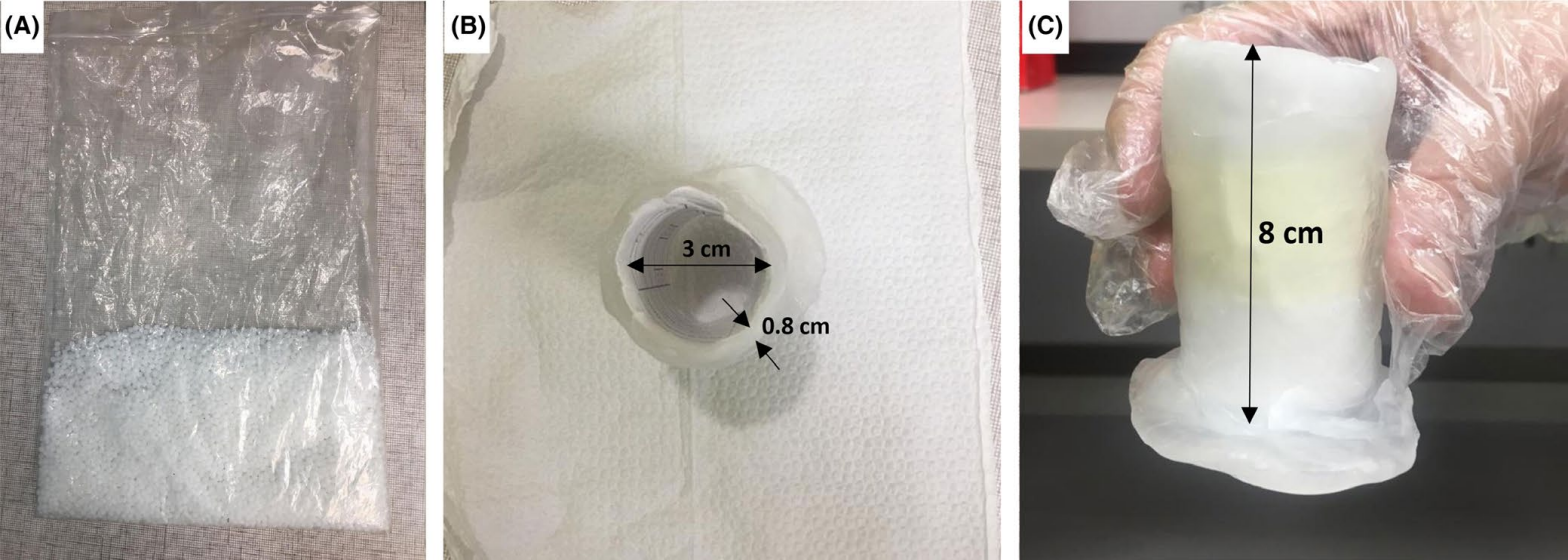
Silicon pellet bolus before soften in water bath (A). Cylindrical holder is made of silicon pellet bolus, (B) top view, and (C) lateral view

The patient underwent CT scan in the frog leg supine position with a slice thickness of 3 mm. For immobilization of penis, we tailored a cylindrical holder based on diameter and length of our patient's penis size to encase the penis vertically. Cylindrical holder is made of silicon pellet bolus (Klarity, Medical Products), as shown in Figure 1A. Silicon pellet bolus is soften in the water bath. Then, we formed it as penis holder. Holder had 8 cm length, 3 cm inner diameter, and 0.8 cm wall thickness (Figure 1B,C).

For reducing radiation dose to the testis, we used a testicular shield in the simulation and treatment fractions. The shield had hemispherical shape with an inner radius of 3.25 cm, a maximum depth of 3.5 cm at the center, and 1.5 cm wall thickness. The inner surface of the shield is coated with a thin layer of dental wax coating to give a smooth surface and reduce backscattered radiation dose from the shield.

**FIGURE 2:**
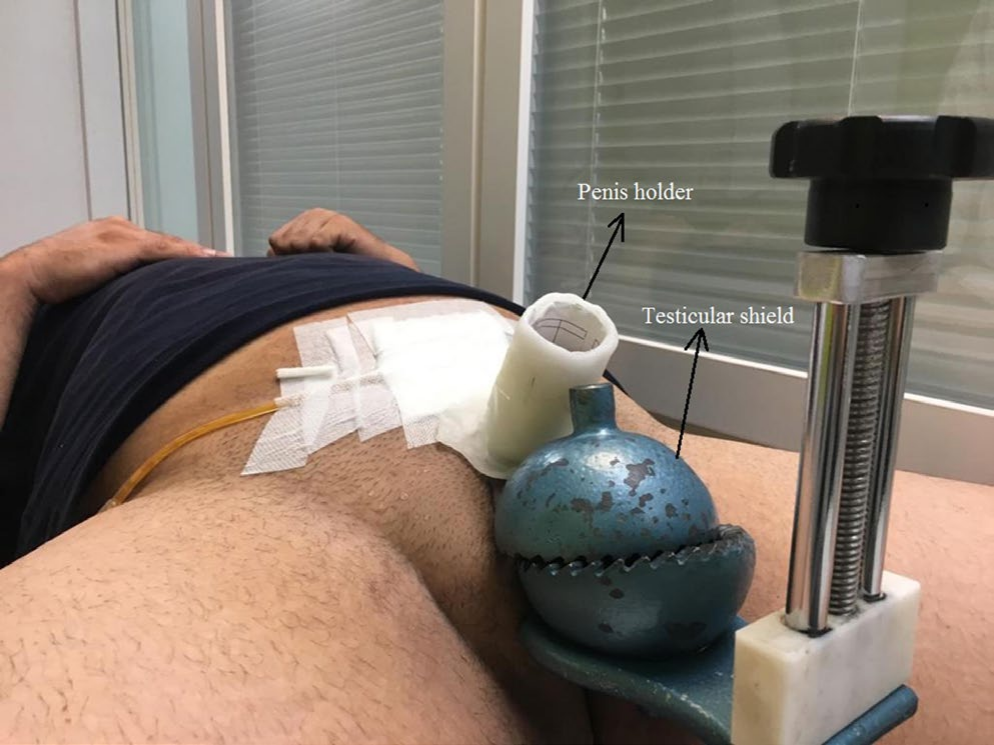
Patient's position for CT simulation, with testicular shield and penis holder in the frog legs position

The patient underwent two consecutive CT scans in the frog leg supine position, with testicular shield and penis holder (as displayed in Figure 2) and then without a testicular shield to remove the metal artifact. A radio‐opaque marker is placed on the patient skin by the responsible physician.

### 2.2 Contouring and treatment planning

Computed tomography datasets were imported into the Eclipse treatment planning system version 13.7 (Varian Medical System). The clinical target volume (CTV) of pelvic lymph nodes (called CTV_LN_) included common iliac, obturator, and external and internal iliac. The high‐risk CTV (CTV_H_) included prostatic and penile urethra. All target volumes were delineated by responsible radiation oncologist on the CT dataset without testicular shield. Also, all contours were rechecked by two radiation oncologists. Planning target volume (PTV) for first phase of radiotherapy was defined as CTV_LN_ plus a 7‐mm margin and CTV_H_ plus a 1‐cm margin. PTV of phase 2 of radiotherapy was defined as CTV_H_ plus a 1‐cm margin. Furthermore, all contours were copied on CT scan with testicular shields and dose calculation was done on both CT scans.

**FIGURE 3:**
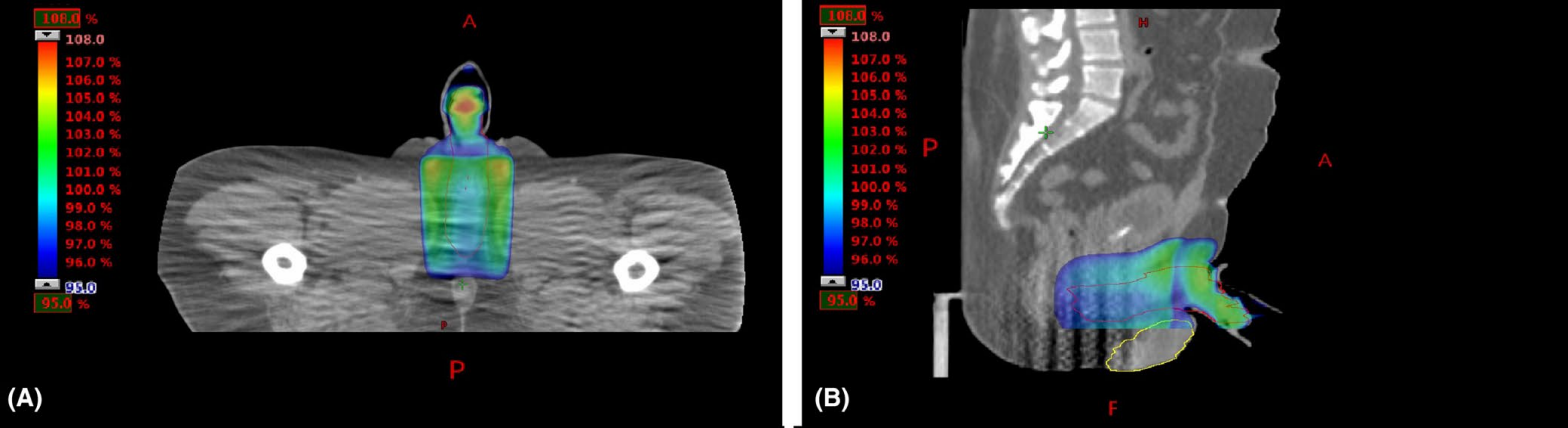
Dose distribution of 95% in axial (A) and sagittal views (B)

We apply the four‐field box technique (field at 0°, 90°, 180°, and 270°). A field in field technique was used to maximize dose coverage of PTV and spare as much as possible the rectum, bladder, and testis. Figure 3 shows the dose distribution in sagittal and axial views.

Prior to each treatment fraction, the patient was positioned at the same position in the CT simulation with penis holder and testicular shield to increase reproducibility of patient position in each fraction and correct position of penile holder marked on skin of patient in first treatment session. Online correction of the pelvic bony marker was performed by an electronic portal imaging device (EPID). Average total time for patient positioning and imaging by EPID before treatment delivery was approximately 6 minutes. The patient had no problem with maintaining stability for the position in simulation and treatment delivery.

## 3 DISCUSSION

This report aimed to propose a new treatment setup for the UC EBRT with reproducible supine technique obtained by homemade and personalized immobilization penis holder, and at the same time, it can reduce radiation dose to testicle using testicular shield. To the best of our knowledge, our reported technique for EBRT of UC has not been previously described in the literature. Different approaches can be used for patient setup during radiotherapy for the penis in the UC.^6^, ^11^ In the traditional setup technique for EBRT of the penile urethra, patient was positioned in the supine position using 10 × 10 cm^2^ wax block that encases penis vertically.^10^, ^11^ Using the aforementioned technique can be more uncomfortable for the patient during EBRT, and also this method does not result in an effective reduction in the mean dose received by testis during radiotherapy course.^11^ In addition, wax block cannot provide a personalized immobilization of the penis owing to the different anatomy of the patients. Another limitation of wax block is lack of availability in some centers.

**FIGURE 4:**
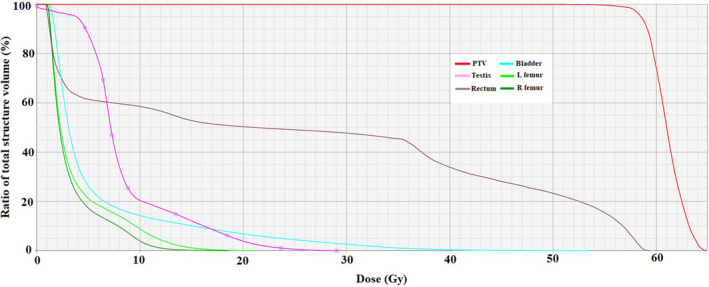
Dose‐volume histogram of PTV and organs at risks

In our proposed technique, we used silicon pellet bolus that is low price material and available in each radiation therapy department. We have built a penis holder with silicon pellet bolus exclusively for each patient based on penile size and diameter, and also we can increase the reproducibility of the patient setup. Another advantage of our method is that a testicular shield can be used at the same time with the penis holder. In addition, dose received by testis was calculated in CT scans with and without testicular shield using Eclipse treatment planning system. The mean dose received by testis with and without the testicular shield was 6.5 Gy and 28 Gy, respectively. Figure 4 shows the organs at risk and PTV dose‐volume histogram with the testicular shield.

Nowadays, three‐dimensional (3D) printing technology has been used in radiation oncology to fabricate personalized and customized treatment accessories such as bolus, brachytherapy applicators, immobilizers, and compensators.^12^, ^13^ Therefore, our proposed immobilization penis holder can be fabricated by 3D printing. Another radiotherapy approach that can be used to treat UC is brachytherapy. Of note, brachytherapy is invasive and has more side effects, as compared to EBRT.^14^

In conclusion, our proposed technique is a novel promising approach that can result in increasing reproducibility of the penis position. It can be also used for other penis malignancies. This report offers an innovative technique for sparing testis in EBRT of UC and penis malignancy, indicating cost‐effectiveness, simplicity, and reproducibility of our method. Larger studies are warranted to evaluate the potential adverse effects associated with this method.

## CONFLICT OF INTEREST

The authors declare that they have no conflict of interests.

## AUTHORS' CONTRIBUTIONS

AR: collected the data, wrote the manuscript, and critically revised the manuscript for important intellectual content.SHM: collected the data and was responsible clinician. BKH and FA: made substantial contributions to conception and design. AT, HZ, AGH, and MA: responsible for the integrity of the work as a whole.

## ETHICAL APPROVAL

This study involved human participants, and it was conducted considering ethic responsibilities according to the World Medical Association and the Declaration of Helsinki. Informed consent was obtained from all individual participants prior to their inclusion in the study.

## RESEARCH INVOLVING HUMAN PARTICIPANTS AND/OR ANIMALS

This study involved human participant, and it was conducted considering ethic responsibilities according to the World Medical Association and the Declaration of Helsinki.

## INFORMED CONSENT

Informed consent was obtained from patient prior to his inclusion in the study.
